# The role of aneuploidy in the emergence of echinocandin resistance in human fungal pathogen *Candida albicans*

**DOI:** 10.1371/journal.ppat.1009564

**Published:** 2021-05-27

**Authors:** Sudisht Kumar Sah, Jeffrey Joseph Hayes, Elena Rustchenko

**Affiliations:** Department of Biochemistry and Biophysics, University of Rochester Medical Center, Rochester, New York, United States of America; Vallabhbhai Patel Chest Institute, INDIA

Fungal diseases largely affect human and animal health and dramatically diminish food crop yields [1]. Among fungi, systemic *Candida* infections are the second or third most common pathogens isolated from blood cultures in the USA [2]. *Candida albicans* is still the predominant *Candida* species, causing up to 50% of candidemia despite an increase in diversity of *Candida* species isolated from clinical samples [3]. In healthy individuals, *C*. *albicans* is a harmless inhabitant of mucosal surfaces throughout the body. However, in immune-compromised individuals, *C*. *albicans* can become a dangerous pathogen, causing severe or even fatal infections. In this review, we summarize recent data linking the reduced susceptibility of *C*. *albicans* cells to mainline echinocandin (ECN) drugs to aneuploidies of chromosomes 5 (Ch5) and Ch2.

## Evolution of ECN resistance and mechanisms influencing susceptibility

The ECN drugs caspofungin, anidulafungin, and micafungin that are recommended as frontline therapy for candidiasis have few adverse actions and drug–drug interactions [4]. ECN drugs kill *C*. *albicans* cells by inhibiting glucan synthase, thus interfering with biosynthesis of the cell wall. Unlike well-studied multiple resistance mechanisms to fluconazole, another common anticandidal from the azole class, there is only one generally recognized mechanism of clinical resistance to drugs from the ECN class. This mechanism involves point mutations in the essential *FKS1* gene (orf19.2929) encoding a catalytic subunit of the 1,3-β-glucan synthase complex. Mutations are clustered in two “hotspot” regions, HS1 and HS2, encompassing residues from 641 to 649, and from 1,345 to 1,365, respectively [5]. Mutations in these regions cause dramatic elevation of *C*. *albicans* minimum inhibitory concentration (MIC) values to ECNs and reduce the sensitivity of the glucan synthase to up to 3,000-fold, the concentration of caspofungin inhibiting 50% of enzymatic activity (IC50) [6].

**Fig 1:**
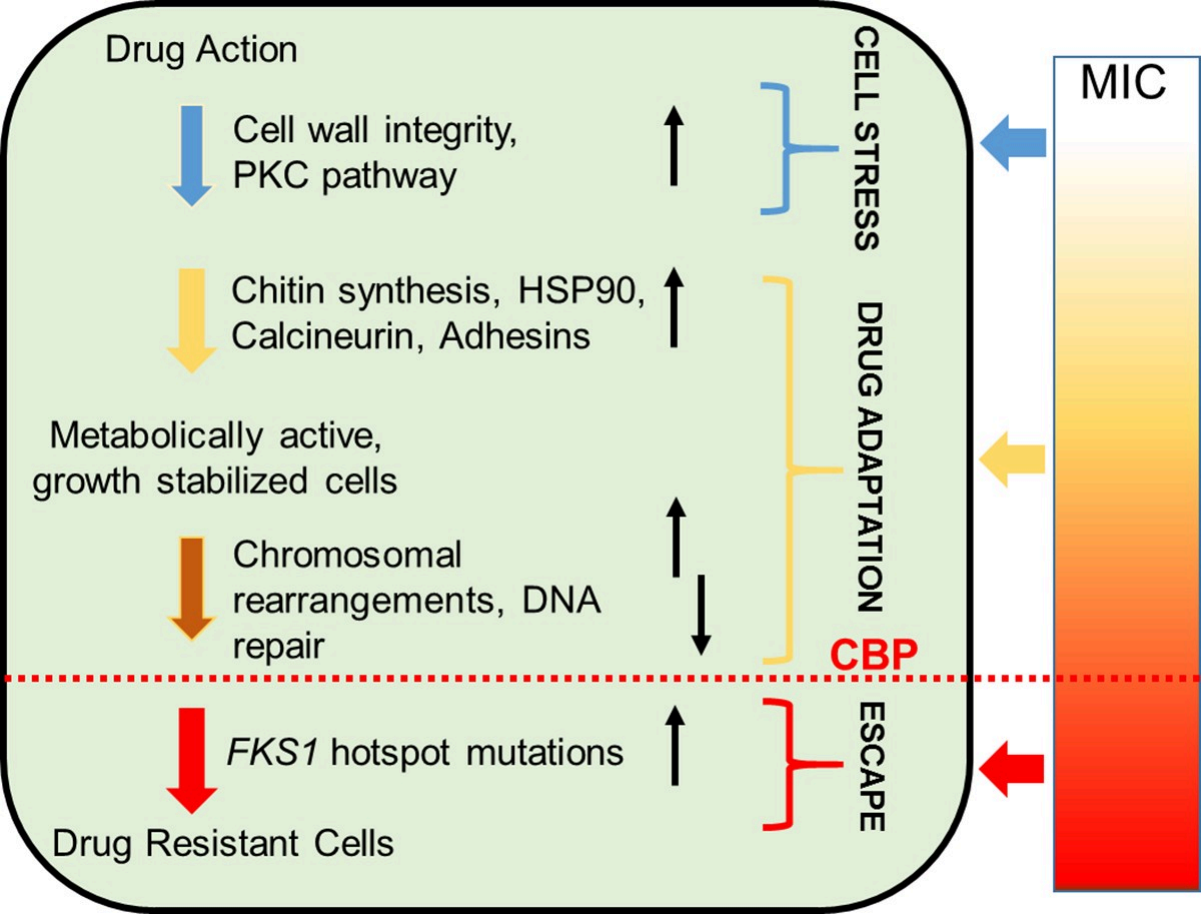
Model presenting multistep evolution of ECN resistance. The CBP of a species is the MIC measured prior to the formation of canonical *FKS1* escape mutations. Increase of MIC is usually observed during drug adaptation. Adapted from [15]. CBP, clinical breakpoint; ECN, echinocandin; HSP90, heat shock protein 90; MIC, minimum inhibitory concentration; PKC, protein kinase C.

However, it has now become obvious that *C*. *albicans* possesses mechanisms independent of *FKS1* mutations that can decrease susceptibility to ECNs, although these “alternative” mechanisms do not confer clinical resistance. These “alternative” mechanisms have been brought to light by dozens of clinical isolates of *Candida* species that display a wide range of increased MIC values for ECNs, including some at or below the MIC breakpoints, but, importantly, without canonical *FKS1* mutations [7–10]. Consistent with these observations, several laboratories found that mutants lacking *FKS1* mutations, but displaying (albeit relatively modest) 2 to 8 fold increases of MIC, can be easily generated in vitro on agar plates supplemented with caspofungin. While *FKS1* mutations leading to resistance can also arise in vitro, these are typically rare [11–13]. Furthermore, while Cowen and colleagues observed evolution of ECN resistance in a series of isogenic clinical isolates of the related species *Candida glabrata*, they demonstrated that mutation to resistance was preceded by a mutation in a different gene that conferred a relatively small increase in MIC [14]. Indeed, quickly arising mutations conferring decreased susceptibility to ECNs are currently viewed as a means to provide a window of opportunity for temporary survival and subsequent formation of resistant *FKS1* mutations. Based on such observations, Healey and Perlin [15] proposed a multistep model in which spontaneously acquired mutations lead to some decrease of drug susceptibility prior to acquisition of *FKS1* resistance mutations and thus play an important role in the evolution of ECN resistance (Fig 1).

Subsequently, given their importance, some of the genes that confer decreased susceptibility have been identified by screening *C*. *albicans* deletion libraries against the ECN caspofungin [16]. In addition, such genes were also identified in mutants that were caspofungin-generated in vitro [17]. Clearly, understanding mechanisms that promote ECN resistance by influencing ECN susceptibility is of high importance. Although currently the incidence of clinical resistance to ECN drugs is relatively low, it is persistent, and the number of resistant cases is growing, primarily due to the increased use of ECNs for disease and prophylactic treatment.

## Spontaneous aneuploidies of *C*. *albicans* control vital functions

*C*. *albicans* has a diploid genome, which is organized into eight pairs of chromosomes that are known for their instability (reviewed in [18]). Aneuploidy, defined as a change in the number of chromosome(s) or large portion of a chromosome, is well tolerated in fungi, including *C*. *albicans*. Spontaneous aneuploidy can be found in populations of *C*. *albicans* cells at high frequencies, between c. 10^−4^ and 10^−2^, with a clear tendency to increase under external stresses, and seems to be a basic property of this microbe [19]. It has been demonstrated that any chromosome of *C*. *albicans* can become aneuploid [18]. Various aneuploidies introduce diversity in a population of cells by controlling vital physiological functions, such as, for example, utilization of different carbon and nitrogen sources [20].

## Specific aneuploidies of *C*. *albicans* Ch5 or Ch2 control adaptation to ECNs

In earlier work, the Rustchenko group demonstrated that *C*. *albicans* employs reversible alterations of specific chromosomes to adapt for growth in the presence of toxic agents that kill cells or prevent cell propagation, including fluconazole, 5-fluoro-orotic acid, and the toxic sugar L-sorbose (Table 1) (reviewed in [18]). Interestingly, adaptation to utilize the secondary sugar D-arabinose also relied on specific alterations of two specific chromosomes [18,21]. Recently, similar experiments demonstrated that reversible aneuploidies of Ch5 or Ch2 can control adaptation to the ECN caspofungin (Table 1) [13,22].

**Table 1:** Specific aneuploidies of Ch5 or Ch2 and corresponding phenotypes.

Chromosome	Alteration	Phenotype	Refs
Ch5	Monosomy	Cas^+^, Sou^+^, 5-Fl^+^, Flu^S^, AmB^S^	[13,23]
	Approximately 395 kb truncation of the right arm adjacent to telomere	Cas^+^, Sou^+^	[17]
	Iso-Ch5R	Cas^+^	[13]
Ch2	Trisomy	Cas^+^, Hu^+^	[22]

The table indicates phenotypes compared to parental diploid strains. Cas^+^, 5-Fl^+^, and Hu^+^ designate reduced susceptibility to caspofungin, 5-fluorocytosin, and hydroxyurea, respectively. Flu^S^ or AmB^S^ designate increased susceptibility to fluconazole or amphotericin B, respectively. Sou^+^ designates resistance to and utilization of toxic sugar L-sorbose.

**Fig 2:**
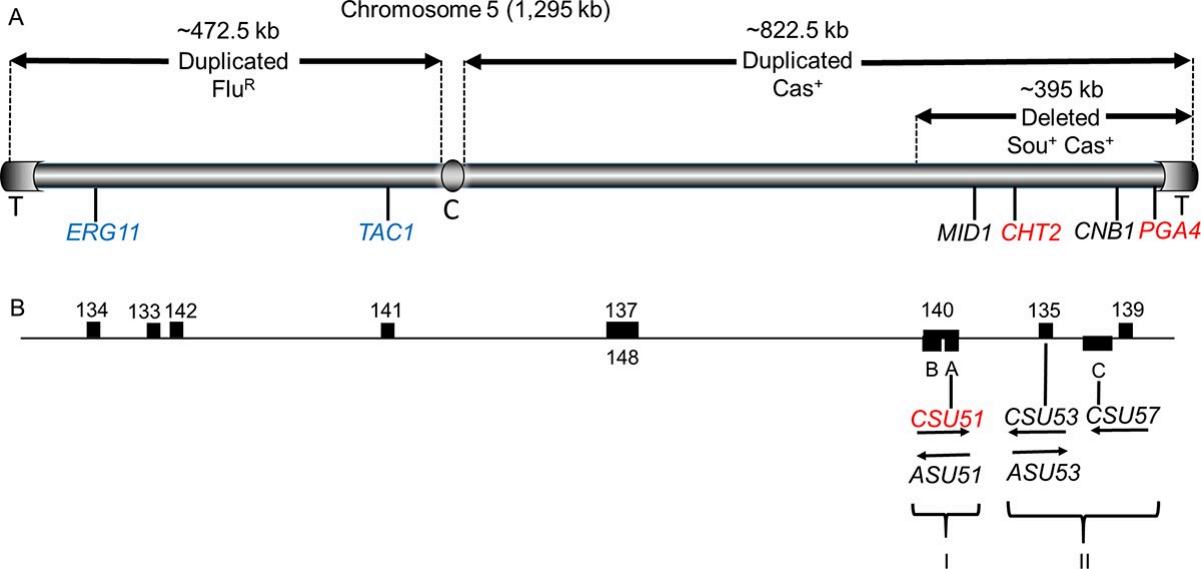
Schematic presentation of Ch5 showing alterations and features that are responsible for different phenotypes. Indicated are: (A) The length (1,295 kb); centromere (C); and telomeres (T). Also shown is the approximately 395 kb truncation of the right arm, and *PGA4*, *CHT2*, *CNB1*, and *MID1* on the right arm, as well as *TAC1* and *ERG11* on the left arm. Note that duplication of the right arm resulting in iso-Ch5R with two right arms confers adaptation to caspofungin, Cas^+^ [13]. While, duplication of left arm resulting in iso-Ch5L with two left arms confers adaptation to fluconazole, Flu^R^ [30]; (B) various regions of Ch5, 133–135, and 137–142 that were cloned from Ch5 library and regions A, B, and C that were identified by their function. Also shown are *CSU51*, *CSU53*, *CSU57* identified in three of the regions and antisense elements *ASU51* and *ASU53* [29,31,32]. Note that genes in red encode negative regulators of ECN susceptibility [17], while genes in blue encode positive regulators of fluconazole susceptibility [33]. Ch5, chromosome 5; ECN, echinocandin.

Loss of one Ch5 results in reduced susceptibility to caspofungin (denoted Cas^+^). In the absence of caspofungin, spontaneous duplication of the remaining Ch5 reverts Cas^+^ cells to the original (parental strain) susceptibility [13] (Table 1). Loss and reduplication of Ch5 also controls resistance to the toxic sugar sorbose (denoted Sou^+^) [24], which kills fungi via a mechanism similar to ECN drugs by inhibiting glucan synthase [25,26]. Interestingly, Ch5 monosomy also confers adaptation to the antifungal pyrimidine analog 5-fluorocytosin (denoted 5-Fl^+^), as well as increased susceptibility to the azole antifungal fluconazole and the polyene antifungal amphotericin B (denoted Flu^S^ and AmB^S^, respectively) [23] (Table 1). Another aneuploid state conferring a Cas^+^ phenotype involves duplication of the right arm of Ch5 to create an isochromosome with two right arms (iso-Ch5R) (Fig 2). Note that these cells carry one iso-Ch5R and one normal Ch5, resulting in three right arms and one left arm of Ch5. Importantly, spontaneous duplication of the remaining normal Ch5 reverts cells to caspofungin susceptibility [13]. In contrast, duplication of the left arm of Ch5 resulting in iso-Ch5L with two left arms confers decreased susceptibility to fluconazole, Flu^R^ (Fig 2), and is found in resistant isolates, as well as in vitro generated mutants [27]. Also, trisomy of Ch2 confers Cas^+^ combined with adaptation to hydroxyurea, Hu^+^ [22]. Of note, genes residing on Ch2 responsible for Cas^+^ phenotype have not yet been identified. However, it is clear that the reversible duplication of Ch2, in contrast to reversible loss of one Ch5, implies that genes for positive regulation of the Cas^+^ phenotype exist on Ch2. It is of interest to this review that exposure of *C*. *albicans* cells to chemotherapeutic drug hydroxyurea results in an Hu^+^ phenotype due to Ch2 trisomy, similarly to exposure to caspofungin [22], the effect, which might contribute to development of *Candida* infection in chemotherapy-treated patient [28]. It is worth mentioning that occasionally a mutant adapts to sorbose via a large deletion within the right arm of Ch5, instead of the loss of an entire chromosome. Consistently, large truncations of the right arm of Ch5 confer both, Sou^+^ and Cas^+^, phenotypes [17,29] (Table 1, Fig 2).

## Complexity of regulation by aneuploidy

Long-term studies of reversible aneuploidy in response to challenge by caspofungin or the toxic sugar sorbose as a model system for *C*. *albicans* adaptation to ECNs have unraveled an astonishingly complex and multilayered regulation of the genes responsible for these phenotypes. These studies show that the rate of formation of (predominantly Ch5 monosomic) Sou^+^ mutants per viable cell per day increased from 10^−6^ at the initial time of detection to 10^−2^ after four days of incubation on selective media [24]. These data suggest that nondisjunction of a Ch5 homologue can occur by different mechanisms and that preexisting and adaptive mutants occur by different processes; the latter ones possibly involving a nonmitotic mechanism. At least nine spatially separated, functionally redundant regions that negatively control resistance to sorbose were identified on Ch5 (Fig 2). These regions, which fall into two functionally redundant pathways I and II (Fig 2), bear no sequence similarity among themselves and four of them bear no similarity to any known sequence. The regions are thought to encompass *CSU* (Control of Sorbose Utilization) genes for negative control of sorbose resistance, three of which *CSU51*, *CSU53*, and *CSU57* were identified in three corresponding regions (Fig 2) [29,31,32]. Most importantly, *CSU51*, a putative transcription factor, negatively controls both ECN susceptibilities and sorbose resistance [17]. It remains to be determined whether the two other known *CSU*s are also implicated with ECNs. The fact that *CSU51* performs this dual function is remarkable, as it opens the possibility that many of the unique *CSU* regulators predicted to reside in different regions of Ch5 also have dual function. Furthermore, two genes, *PGA4* and *CHT2*, which participate in cell wall construction and encode negative regulators of only ECN susceptibilities, have also been identified (Fig 2) [17]. However, the final number of either *CSU*s or genes involved only in ECN susceptibility remains elusive.

It is tempting to explain the Cas^+^ phenotype resulting from Ch5 monosomy as due to diminished gene dose of multiple negative regulators scattered across this chromosome. However, the fact that the formation of iso-Ch5R, resulting in three right arms versus one left arm of Ch5, also results in diminished ECN susceptibility in corresponding cells implies that an additional or more complicated scenario can be in play. The right arm of Ch5 carries two genes, *MID1* and *CNB1*, which encode positive regulators of ECN susceptibility [34,35]. Therefore, it is possible that the effects of various Ch5 ploidies are due to a balance between negative and positive factors expressed from this chromosome. From this point of view, the loss of one Ch5 diminishes the action of both negative and positive regulators expressed from this chromosome; negative regulators overriding positive regulators. Whereas, in the condition of iso-Ch5R, the amplified positive regulators on the right arm of Ch5 override the action of negative regulators on this arm.

As an example, Ch5 contains two key genes of interest, *TAC1*, a positive regulator of *CDR1* and *CDR2* genes encoding fluconazole efflux pumps on Ch3, and *ERG11*, a target gene for fluconazole (Fig 2A) [27]. Thus, loss of one Ch5, diminishing the copy number of *TAC1* and *ERG11*, likely leads to increased sensitivity to fluconazole via loss of activity of these efflux pumps and the fluconazole target. In addition, as loss of one Ch5 also increases sensitivity to amphotericin B (see Table 1), it may be expected that Ch5 also carries genes for positive regulation of susceptibility to this drug. On a contrary, negative regulator(s) of antifungal 5-fluorocytosin could be also expected to reside on Ch5, as cells with monosomic Ch5 acquire 5-Fl^+^ phenotype (see Table 1).

An additional level of regulation includes regulatory elements denoted *ASU* (Antisense regulators of Sorbose Utilization) that are embedded within *CSU*s in an antisense configuration (Fig 2). In respect to the *CSU* transcripts, the *ASU* long noncoding transcripts are completely overlapped by *CSU* transcripts, are in lesser amounts, and are inversely related. Presumably, *ASU* transcripts modulate *CSU* transcripts [31]. Some genes residing on aneuploid chromosomes are also controlled by transcriptional compensation for gene dosage, which keeps expression of select genes at or near the diploid level, irrespectively of chromosome ploidy [36,37]; *MID1* and *CNB1* exemplifying such genes (Fig 2) [13]. Indeed, widespread dosage compensation occurs across monosomic Ch5 and correlates with increased chromosome-wide acetylation of histone H4 [38]. This epigenetic feature involves the histone acetyltransferase complex NuA4, which could be a novel drug target to reduce the viability of resistant cells. On the other hand, decreased expression of some genes to the diploid level has been shown to occur within the trisomic Ch4/7 and correlates with increased acetylation of histone H3, but the histone acetyltransferases involved have not been identified [38].

Last, but not least, the *C*. *albicans* genome contains two genes, *FKS2* and *FKS3*, which have considerable sequence identity with the key ECN resistance gene *FKS1* but act as negative regulators of *FKS1* [39]. While heterozygous deletion of the essential *FKS1* gene results in increased ECN susceptibility, complete removal of either *FKS2* or *FKS3* results, in contrast, in decreased ECN susceptibility due to resultant overexpression of *FKS1*, leading to an increase in cell wall glucan. Other indications of the involvement of *FKS2* and *FKS3* in ECN susceptibility include wide variations in the expression levels of these genes relative to *FKS1* in clinical resistant isolates, down-regulation in spontaneous laboratory mutants harboring *FKS1* resistance mutations [40], as well as down-regulation in model mutants bearing monosomic Ch5 or iso-Ch5R but lacking *FKS1* resistance mutations [13].

ECN susceptibility in *C*. *albicans* can be diminished by a limited number of distinct aneuploidies of Ch5 and Ch2, which is consistent with an earlier assumption that genes of *C*. *albicans* are distributed over chromosomes nonrandomly [29,41]. A number of genes and processes relevant to this control already have been elucidated and inform about much-needed potential drug targets. However, we are clearly at the beginning of an exciting journey into understanding the regulation of ECN drug susceptibility by chromosome aneuploidy, which involves a complex interplay between ratio of negative and positive regulators on Ch5 and Ch2, the regulation of *FKS* genes residing outside Ch5 and Ch2, additional factors involved in the complex regulation of genes on aneuploid chromosomes, and still unidentified genes and features. A better understanding of the control exerted by aneuploidies will help to better understand evolution of ECN drug resistance and will facilitate the identification of new drug targets.
